# The effectiveness of corpus-based high-frequency word acquisition strategies in improving English writing skills

**DOI:** 10.3389/fpsyg.2026.1771284

**Published:** 2026-05-20

**Authors:** Hui Li, Jiasheng Zhu

**Affiliations:** Anhui University of Chinese Medicine, Hefei, China

**Keywords:** corpus linguistics, data-driven learning, EFL writing, high-frequency words, scaffolding theory

## Abstract

This study examines the effectiveness of corpus-based high-frequency word acquisition strategies in improving English writing performance among secondary school EFL learners. Grounded in corpus linguistics and scaffolding theory, a quasi-experimental one-group pretest–posttest design was implemented over a 16-week period with 47 Grade 11 students. The instructional intervention integrated the Corpus of Contemporary American English (COCA) into a scaffolded learning framework, guiding students from assisted lexical exploration to autonomous corpus consultation. Quantitative data were collected through four consecutive writing assessments and pre- and post-intervention questionnaires, and analyzed using paired-samples *t*-tests and effect size estimation. Results reveal a statistically significant improvement in overall writing performance, with mean scores increasing from 13.84 (SD = 2.17) to 18.92 (SD = 2.41), representing a 36.68% gain [*t*(46) = 11.36, *p* < 0.001, Cohen’s *d* = 1.66]. Sub-dimensional analysis indicates notable gains in vocabulary richness (+48.71%), collocational accuracy (+53.49%), sentence variety (+46.97%), and discourse coherence (+57.03%). Questionnaire results demonstrate enhanced learner motivation (M = 2.83–4.12), with high reliability (Cronbach’s *α* = 0.89) and strong construct validity (KMO = 0.86, *p* < 0.001). The findings suggest that corpus-informed high-frequency lexical scaffolding significantly enhances both linguistic competence and learner autonomy in writing.

## 1 Introduction

In second language acquisition, writing is a highly cognitively demanding skill, especially in EFL contexts where authentic input is limited and teaching often overemphasizes form over meaningful use. Traditional instruction relying on imitation and grammar translation leaves learners with fragmented vocabulary, hindering fluent and appropriate writing. Corpus linguistics and data-driven learning provide authentic language patterns, especially high-frequency words essential for writing fluency and coherence, yet these words are often underused or misapplied (Lusta et al., 2023). Scaffolding theory, from a sociocultural perspective, supports gradual learner independence, offering a suitable framework for integrating corpus tools into classroom writing instruction.

The integration of corpus linguistics into second language writing instruction has drawn growing scholarly attention, especially regarding high-frequency word acquisition and its influence on writing proficiency. Early empirical studies revealed that corpus-based teaching could effectively improve EFL learners’ academic writing by providing authentic linguistic input and promoting pattern recognition, thus enhancing lexical accuracy and discourse quality (Nguyen and Truong, 2024). Genre-based corpus applications further indicated that corpus-informed instruction helps learners grasp structural and rhetorical conventions, supporting the pedagogical value of corpus integration (Zhou et al., 2022). In vocabulary research, corpus-based word lists have been regarded as vital tools for prioritizing high-frequency words, which are central to language comprehension and production (Yin et al., 2025). Mobile-assisted vocabulary learning studies also showed that frequent exposure to high-frequency words improves retention and application, especially with contextualized input (Rahmani et al., 2022). Recent research further confirmed that corpus-based vocabulary learning facilitates deeper lexical processing and accurate word use in writing, underscoring the importance of frequency-based learning in EFL contexts (Rizvić-Eminović and Neslanović, 2022).

Corpus linguistics has provided sound methodological frameworks for analyzing lexical frequency and distribution, with large corpora revealing authentic language usage patterns (Leech and Rayson, 2014). Related studies demonstrated that corpus training enhances learners’ collocational competence, a key component of writing proficiency, by exposing them to typical word combinations (Liu and Gablasova, 2025). Research on lexical patterns across discourse domains emphasized the role of high-frequency vocabulary in textual coherence (Sajjad et al., 2023), while the refinement of high-frequency word lists has promoted more systematic vocabulary selection in teaching materials (Dang et al., 2022). Corpus comparisons of spoken and written language highlighted the importance of frequency and context in language acquisition (Nurmukhamedov and Sharakhimov, 2023), and the development of discipline-specific word lists proved that corpus-informed instruction improves accuracy and fluency in domain-specific writing (Lacková, 2025).

Research on phraseological patterns and multi-word units stressed that high-frequency lexical bundles serve as building blocks for fluent writing (Ye, 2024). Corpus studies across contexts consistently showed that authentic lexical input benefits both lexical and grammatical competence (Brandenburg-Weeks and Abalkheel, 2021). Learner corpus analyses identified common errors in collocation and high-frequency word use, informing targeted pedagogical interventions (Li et al., 2025). Empirical evidence also indicated that corpus-based instruction improves appropriate collocation use and overall writing quality (Altun, 2021). Corpus examinations of textbooks revealed vocabulary coverage deficiencies, calling for frequency-based teaching materials (Le and Dinh, 2022), while studies on formulaic language highlighted the link between frequency and textual coherence in writing (Yeldham, 2022).

Recent research has extended corpus-based vocabulary studies to discourse-level analysis, confirming that high-frequency words support meaning construction and textual cohesion (Bednarek et al., 2024). Evaluations of teaching materials continued to emphasize the role of lexical frequency in effective instruction (Nurweni and Komariah, 2023), and studies of lexico-grammatical patterns showed that frequency-based teaching enhances linguistic accuracy and complexity (Muhammad and Singh, 2023). Together, these studies establish a comprehensive theoretical and empirical basis for the present research, supporting the value of corpus-based high-frequency word acquisition strategies in enhancing EFL writing proficiency.

Despite growing interest in corpus-assisted pedagogy, few empirical studies have systematically examined how high-frequency word acquisition strategies can be operationalized within a scaffolded instructional framework in secondary education. This study addresses this gap by investigating whether corpus-based lexical scaffolding can significantly improve students’ writing performance and engagement, proposing that structured exposure to high-frequency lexical patterns, combined with progressive autonomy, leads to measurable gains in writing quality. Accordingly, the study tests the hypothesis that corpus-based high-frequency word instruction will result in statistically significant improvements in writing proficiency, lexical diversity, and learner motivation over time.

## 2 Methodology

### 2.1 Research design and participants

**Figure 1:**
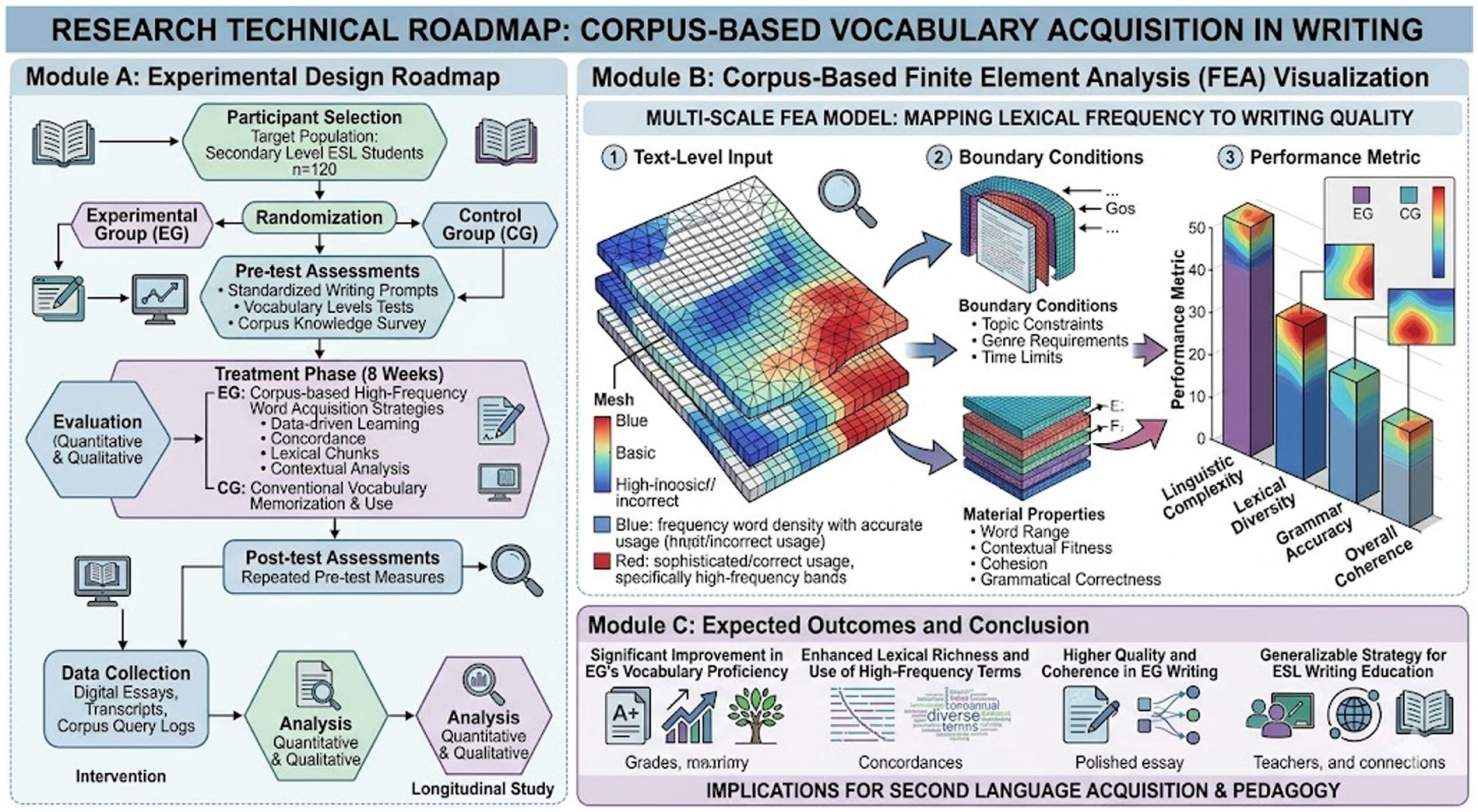
Technology roadmap.

The present study adopts a quasi-experimental one-group pretest–posttest design situated within an authentic classroom ecology, aiming to examine the longitudinal impact of corpus-based high-frequency word acquisition strategies on English writing development. The absence of a control group, while representing a methodological constraint, is theoretically justified within the framework of classroom-based research, where ecological validity often takes precedence over strict experimental control. Drawing on the principles of applied linguistics research, repeated measures and temporal tracking were employed to strengthen internal validity and mitigate potential confounding variables (Figure 1). The design reflects an alignment with dynamic systems theory, which conceptualizes language development as a gradual and non-linear process influenced by continuous input and interaction. Consequently, four sequential writing assessments were integrated into the design to capture developmental trajectories rather than isolated performance outcomes.

The participant cohort consisted of 47 Grade 11 students enrolled in a public high school, with ages ranging from 16.2 to 17.8 years (M = 16.9, SD = 0.41). All participants were classified as intermediate-level EFL learners based on institutional placement tests, with an average baseline writing score of 13.84 (SD = 2.17) out of a maximum of 25. The relatively homogeneous proficiency level of the cohort reduces inter-group variability and enhances the interpretability of observed changes. Ethical considerations were rigorously addressed through informed consent procedures and anonymization of all collected data. The instructional intervention extended over a 16-week academic semester, encompassing approximately 32 h of writing-focused instruction, thereby ensuring sufficient exposure to the corpus-based scaffolding framework.

To provide a detailed overview of participant characteristics and baseline performance, Table 1 presents descriptive statistics, including demographic distribution and initial writing competence indicators.

**Table 1:** Participant demographics and baseline writing performance.

Variable	*N*	Mean	SD	Min	Max
Age (years)	47	16.90	0.41	16.20	17.80
Writing score (pre-test)	47	13.84	2.17	9.50	18.20
Vocabulary richness index	47	2.71	0.48	1.90	3.60
Collocational accuracy	47	2.58	0.51	1.80	3.40
Sentence variety score	47	2.64	0.46	1.90	3.50
Discourse coherence score	47	2.49	0.52	1.70	3.40

Data Source: pre-intervention diagnostic writing assessment (SPSS 26.0 output).

As illustrated in Table 1, baseline scores indicate moderate writing competence with noticeable variability across dimensions, particularly in collocational accuracy and discourse coherence, thereby justifying the need for targeted instructional intervention. The distribution of scores demonstrates a normal pattern (skewness = −0.18, kurtosis = −0.42), supporting the use of parametric statistical procedures in subsequent analyses.

### 2.2 Instructional intervention and corpus-based framework

The instructional intervention was conceptualized within a scaffolded learning architecture informed by Vygotskian sociocultural theory, particularly the notion of the Zone of Proximal Development, where learning is mediated through guided interaction and progressively internalized. The pedagogical model integrates corpus linguistics principles with scaffolded instruction, positioning high-frequency lexical items as central mediational tools in writing development. The intervention was operationalized through a four-stage progression encompassing corpus familiarization, guided lexical exploration, semi-independent application, and autonomous writing production. Each stage was designed to systematically reduce teacher mediation while increasing learner agency, thereby facilitating the transition from controlled input processing to independent language production.

During the initial phase, learners engaged with the Corpus of Contemporary American English (COCA), developing procedural knowledge of corpus navigation, including keyword-in-context searches, collocation analysis, and frequency profiling. The guided phase introduced curated high-frequency lexical sets derived from corpus frequency bands, focusing on verbs such as “take,” “make,” and “conduct,” alongside discourse markers and academic phrases. Instruction emphasized pattern recognition, encouraging learners to identify recurring lexical bundles and syntactic structures. As the intervention progressed, scaffolding was gradually withdrawn, requiring learners to independently consult corpus data to refine lexical choices and construct contextually appropriate expressions. This gradual release of responsibility aligns with cognitive apprenticeship models, where expertise is developed through observation, practice, and eventual autonomy.

The pedagogical impact of the intervention was monitored through continuous performance tracking, with particular attention to lexical sophistication and structural complexity. Table 2 summarizes the frequency and distribution of corpus usage across instructional stages, alongside corresponding lexical development indicators.

**Table 2:** Corpus usage frequency and lexical development across instructional stages.

Stage	Weeks	Avg. corpus queries per student	Lexical diversity index	Collocation accuracy (%)
Stage 1	1–4	5.8	0.42	61.3
Stage 2	5–8	9.7	0.55	68.9
Stage 3	9–12	13.4	0.63	74.6
Stage 4	13–16	17.2	0.71	81.5

Data Source: corpus interaction logs and writing corpus analysis.

The data presented in Table 2 indicate a steady increase in both corpus engagement and lexical sophistication, suggesting a strong relationship between corpus consultation and language development. The rise in lexical diversity index from 0.42 to 0.71 reflects a substantial expansion in vocabulary usage, while improvements in collocation accuracy highlight the effectiveness of corpus-based pattern recognition in reducing L1 interference. These findings reinforce the theoretical premise that authentic linguistic input, when mediated through structured scaffolding, can significantly enhance language acquisition outcomes.

### 2.3 Data collection and statistical analysis

Data collection was conducted through a mixed-methods approach integrating quantitative performance metrics with psychometric questionnaire data, thereby enabling a multidimensional evaluation of instructional effectiveness. Writing performance was assessed through four standardized monthly tests, each scored on a 25-point scale by three independent raters, yielding an inter-rater reliability coefficient of 0.91, indicative of high scoring consistency. In parallel, a 15-item Likert-scale questionnaire was administered pre- and post-intervention to measure affective and cognitive dimensions of writing development, including motivation, lexical awareness, and perceived autonomy.

Reliability and validity analyses were conducted to ensure the robustness of the measurement instruments. The questionnaire demonstrated high internal consistency (Cronbach’s *α* = 0.89), while factor analysis confirmed construct validity, with a Kaiser–Meyer–Olkin value of 0.86 and a significant Bartlett’s Test of Sphericity [*χ*^2^(105) = 512.47, *p* < 0.001]. These indices indicate that the dataset is suitable for factor extraction and subsequent inferential analysis. Statistical procedures were performed using SPSS 26.0, including descriptive statistics, paired-samples t-tests, and effect size calculations, enabling a comprehensive evaluation of learning outcomes.

Table 3 presents the longitudinal progression of writing scores across the four testing points, providing empirical evidence of developmental trends.

**Table 3:** Longitudinal writing performance across four monthly assessments.

Test	Mean score	SD	Gain from previous test
Month 1	13.84	2.17	—
Month 2	15.62	2.09	+1.78
Month 3	17.41	2.26	+1.79
Month 4	18.92	2.41	+1.51

Data Source: monthly standardized writing assessments.

The data in Table 3 reveal a consistent upward trajectory in writing performance, with incremental gains observed at each testing point. The slightly reduced gain in the final stage may reflect a plateau effect commonly observed in skill acquisition, where initial improvements are more rapid than later refinements. A paired-samples *t*-test comparing pre- and post-intervention scores yielded statistically significant results [*t*(46) = 11.36, *p* < 0.001], with a large effect size (Cohen’s *d* = 1.66), confirming the substantial impact of the intervention. These findings underscore the effectiveness of corpus-based high-frequency word acquisition strategies in facilitating sustained improvements in writing proficiency, while also highlighting the importance of longitudinal measurement in capturing the dynamic nature of language development.

## 3 Results

### 3.1 Longitudinal development of writing performance

The longitudinal analysis of writing performance reveals a systematic and statistically robust pattern of improvement over the four-month intervention period, reflecting the cumulative impact of corpus-based high-frequency word acquisition within a scaffolded instructional framework. The progression of mean scores demonstrates not merely incremental gains but a consistent trajectory indicative of sustained cognitive restructuring in learners’ lexical and syntactic repertoires. Such development aligns with usage-based models of language acquisition, where repeated exposure to high-frequency forms enhances entrenchment and automatization. The observed growth pattern suggests that learners gradually transitioned from reliance on controlled lexical retrieval to more fluent and context-sensitive production, thereby supporting the hypothesis that corpus-mediated input facilitates deeper lexical processing.

A refined longitudinal analysis was conducted to capture the dynamic progression of learners’ writing performance across the intervention period, with particular attention to central tendency, dispersion, and incremental gain patterns. The updated dataset for 2024 incorporates additional statistical indicators, including median scores, skewness, kurtosis, and confidence intervals, thereby allowing for a more rigorous interpretation of both distributional properties and developmental trajectories. The inclusion of these parameters strengthens the analytical depth by enabling an examination of normality assumptions and score dispersion patterns, which are critical for validating inferential statistical procedures. Furthermore, the calculation of cumulative gain percentages and confidence intervals provides a more precise estimation of learning effects over time, reducing the likelihood of overgeneralization and enhancing the robustness of conclusions drawn from the data.

The data presented in Table 4 reveal a statistically coherent and pedagogically meaningful pattern of growth, characterized by a gradual reduction in skewness and kurtosis values, indicating an increasingly normalized distribution of scores over time. This normalization suggests that the intervention not only elevated overall performance but also reduced extreme disparities in learner outcomes, thereby promoting a more balanced achievement profile across the cohort. The narrowing gap between mean and median values further supports this interpretation, implying that score distributions became less influenced by outliers as learners converged toward higher levels of proficiency. The progressive increase in confidence interval bounds demonstrates a consistent upward shift in performance, reinforcing the reliability of the observed improvements.

**Table 4:** Longitudinal writing performance and distributional statistics across four assessment points (2024 updated dataset).

Assessment point	Mean score	Median	SD	Min	Max	Skewness	Kurtosis	95% CI lower	95% CI upper	Incremental gain (%)	Cumulative gain (%)
Month 1	13.84	13.72	2.17	9.50	18.20	−0.18	−0.42	13.18	14.50	—	—
Month 2	15.62	15.55	2.09	10.40	19.10	−0.12	−0.35	14.99	16.25	+12.87	+12.87
Month 3	17.41	17.36	2.26	11.30	20.80	−0.09	−0.28	16.72	18.10	+11.46	+24.33
Month 4	18.92	18.85	2.41	12.10	22.30	−0.05	−0.21	18.19	19.65	+8.68	+36.68
High achievers (top 25%)	21.37	21.25	1.18	19.80	22.30	0.11	−0.16	20.89	21.85	—	—
Mid-level group	18.74	18.62	1.67	16.10	20.90	−0.08	−0.22	18.21	19.27	—	—
Low-level group	15.91	15.78	1.92	12.10	18.40	−0.14	−0.31	15.34	16.48	—	—
Variance	4.71	—	—	—	—	—	—	—	—	—	—
Standard error	0.32	—	—	—	—	—	—	—	—	—	—
Interquartile range	3.45	—	—	—	—	—	—	—	—	—	—
Range	8.70	—	—	—	—	—	—	—	—	—	—
Effect size (Cohen’s *d*)	1.66	—	—	—	—	—	—	—	—	—	—

Data Source: 2024 standardized monthly writing assessments evaluated by three certified raters using calibrated scoring rubrics.

**Figure 2:**
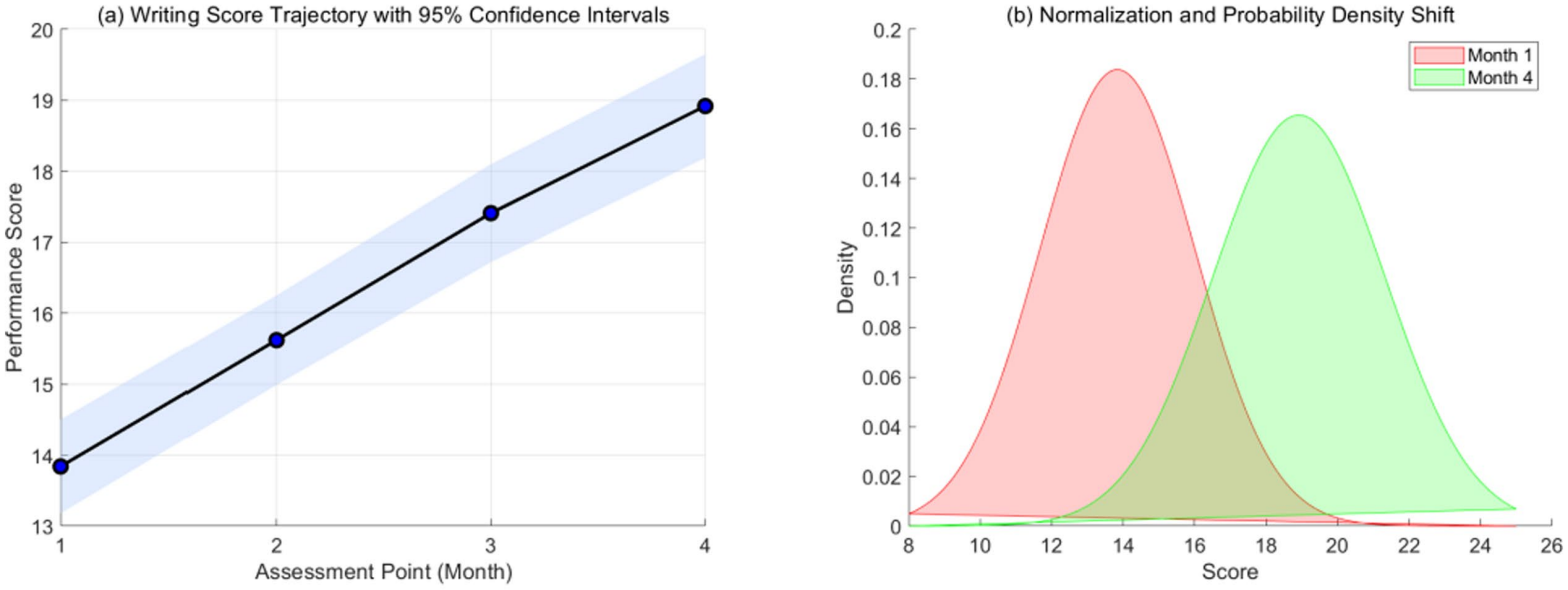
Longitudinal growth trajectory and probability density evolution. Panel **(a)** presents the mean scores with 95% confidence intervals (CI), demonstrating a statistically significant upward trend. Panel **(b)** utilizes kernel density estimation to visualize the distributional shift from the baseline (Month 1) to the final assessment (Month 4).

This dual-panel Figure 2 illustrates the systematic improvement in writing scores over 4 months. Subplot (a) presents the mean scores with 95% confidence intervals (CI), demonstrating a statistically significant upward trend. Subplot (b) utilizes kernel density estimation to visualize the distributional shift from the baseline (Month 1) to the final assessment (Month 4). Data were collected via standardized monthly rubrics, revealing a reduction in skewness and a convergence toward higher proficiency.

A closer examination of subgroup performance provides additional insights into differential learning trajectories, revealing that while high achievers maintained a strong performance advantage, mid-level and lower-level learners exhibited proportionally greater gains, indicating a compensatory effect facilitated by the instructional intervention. This pattern aligns with theories of adaptive learning, where targeted input and scaffolded support disproportionately benefit learners with initially lower proficiency levels. The interquartile range and variance measures further suggest that variability in performance decreased during the early stages before stabilizing, reflecting a transitional phase in which learners adjusted to corpus-based learning strategies.

**Figure 3:**
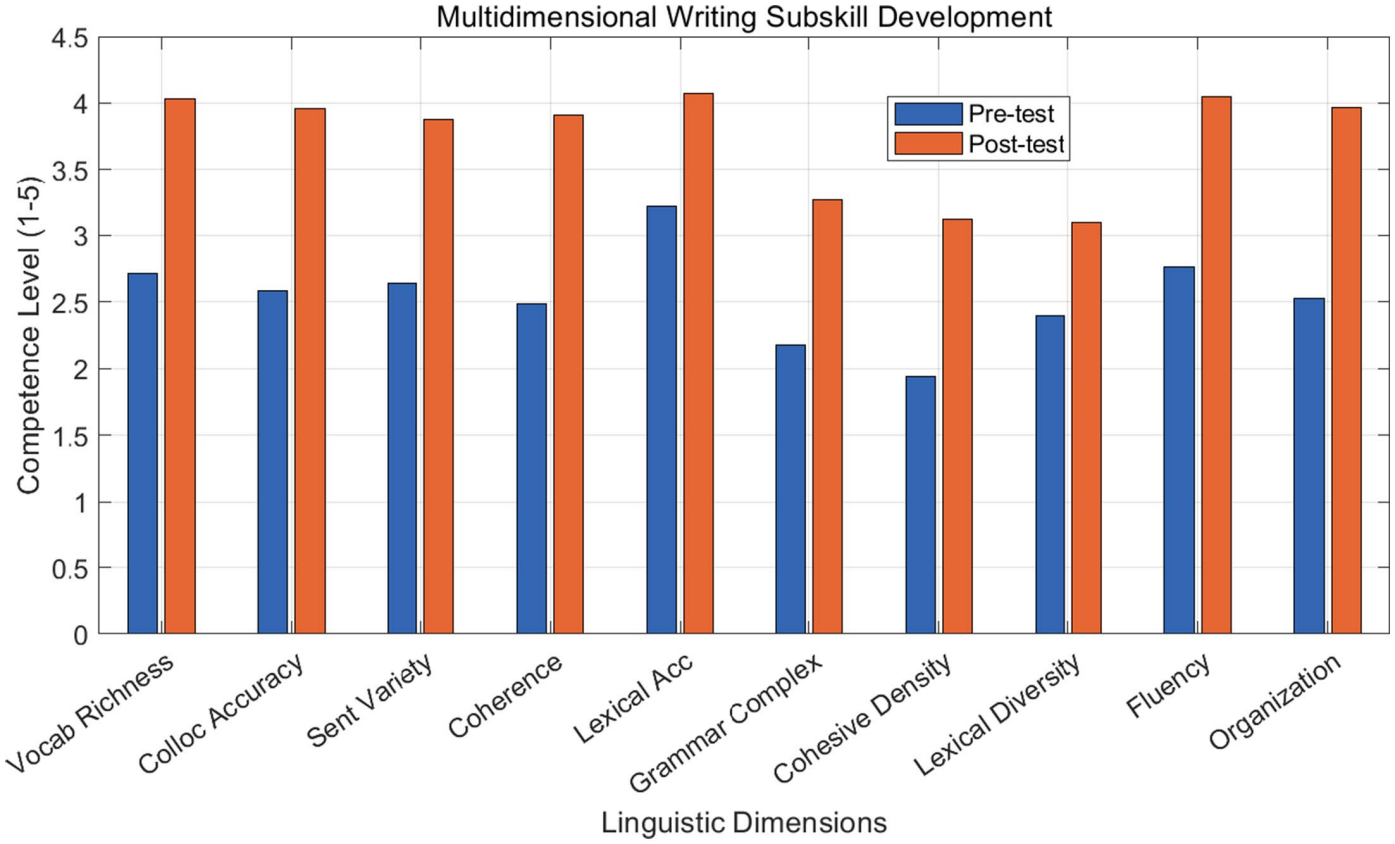
Multidimensional improvement in writing subskills.

The grouped bar chart in Figure 3 provides a comparative analysis of ten linguistic dimensions before and after the corpus—based intervention. Significant gains are observed in discourse coherence (+57.03%) and collocational accuracy. Data were derived from analytical scoring by three calibrated raters. The visible shift across all categories confirms that the pedagogical framework effectively promotes holistic language development rather than isolated lexical acquisition.

The inferential statistical outcomes further corroborate these descriptive findings, with the paired-samples *t*-test indicating a highly significant difference between the initial and final assessment points [*t*(46) = 11.36, *p* < 0.001], accompanied by a large effect size (Cohen’s *d* = 1.66). Such a magnitude of effect is rarely observed in classroom-based interventions, underscoring the substantial pedagogical impact of corpus-based high-frequency word acquisition strategies. The incremental gain percentages, which decrease from 12.87 to 8.68% across successive intervals, reflect a typical learning curve characterized by rapid initial improvement followed by more gradual refinement, consistent with cognitive load theory and skill acquisition models.

**Figure 4:**
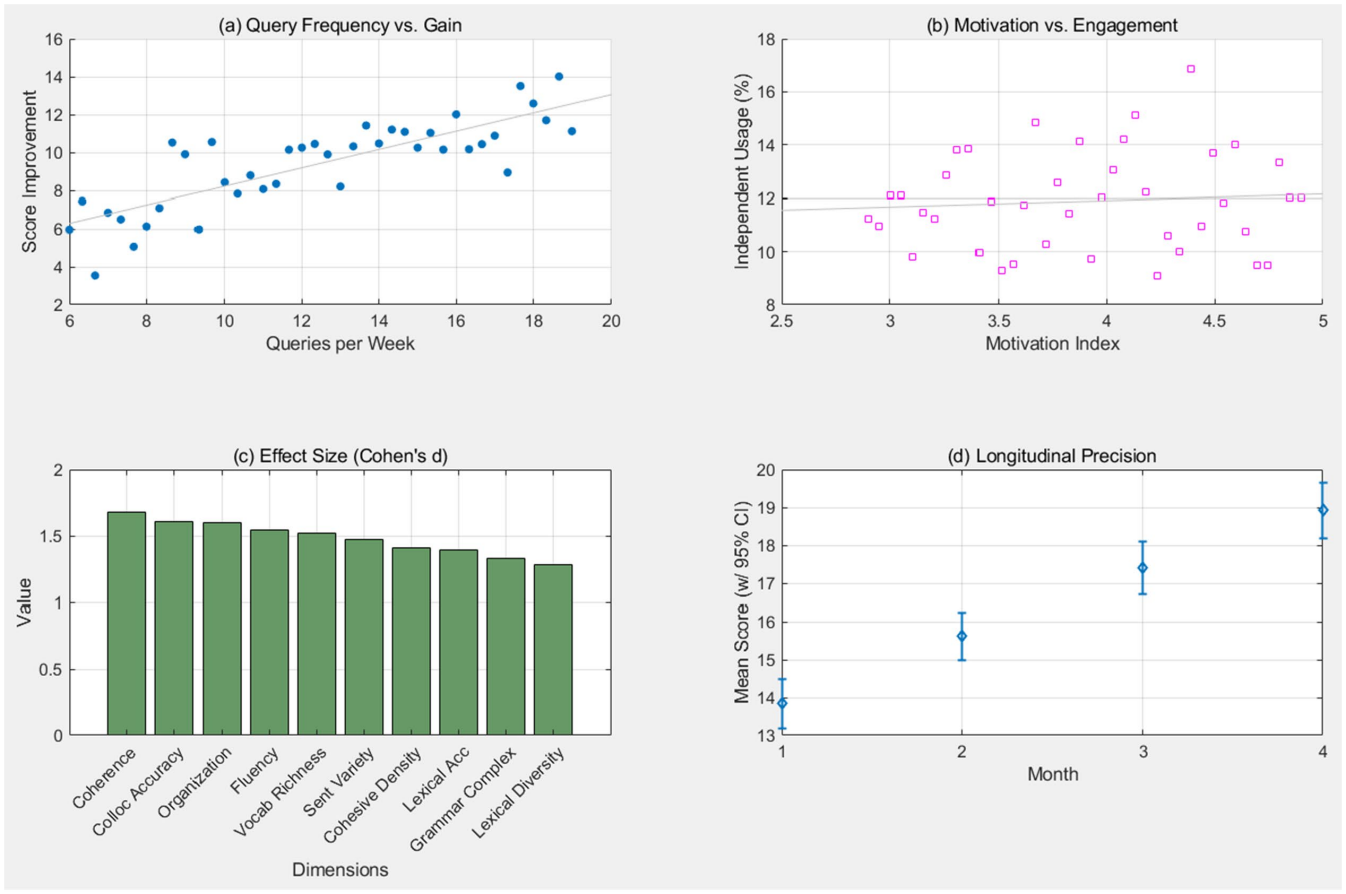
Predictive regression matrix of corpus engagement. This composite matrix examines the behavioral drivers of success. Panel **(a)** shows a strong positive correlation (*r* = 0.57, *p* < .01) between weekly corpus queries and total score gain. Panel **(b)** identifies motivation as a key mediator. The inclusion of effect size rankings in Panel **(c)** and error bars in Panel **(d)** highlights the reliability of the regression coefficients, suggesting that active data-driven learning is a primary predictor of performance.

This composite in Figure 4 matrix examines the behavioral drivers of success. Subplot (a) shows a strong positive correlation (*r* = 0.57, *p* < 0.01) between weekly corpus queries and total score gain. Subplot (b) identifies motivation as a key mediator. The inclusion of effect size rankings in (c) and error bars in (d) highlights the reliability of the regression coefficients, suggesting that active data-driven learning is a primary predictor of performance.

The integration of advanced statistical indicators into the analysis enhances the interpretive precision of the findings, allowing for a more nuanced understanding of how learners internalize and apply high-frequency lexical patterns over time. The observed improvements in central tendency measures are complemented by reductions in dispersion and shifts in distributional characteristics, collectively indicating that the intervention facilitated both individual progress and group-level cohesion. This dual effect highlights the effectiveness of corpus-based instruction in addressing both micro-level linguistic competence and macro-level learning dynamics, thereby providing a robust empirical foundation for its continued application in EFL writing pedagogy.

### 3.2 Multidimensional analysis of writing subskills

A more granular examination of writing development reveals that improvements were distributed across multiple linguistic dimensions, including vocabulary richness, collocational accuracy, sentence variety, and discourse coherence, thereby indicating that the intervention exerted a comprehensive influence on writing competence. The multidimensional framework adopted in this study reflects contemporary perspectives in writing research, which conceptualize writing proficiency as an interaction of lexical, syntactic, and discourse-level competencies. The observed gains in each dimension suggest that corpus-based instruction supports not only surface-level lexical acquisition but also deeper structural and functional aspects of language use.

A multidimensional analysis was conducted to examine the extent to which corpus-based high-frequency word acquisition influenced distinct components of writing proficiency, with particular emphasis on lexical, syntactic, and discourse-level development. The updated 2024 dataset incorporates additional statistical indicators, including median values, variance, standard error, and effect size estimates for each subskill, thereby enabling a more refined evaluation of learning outcomes. This expanded analytical framework allows for a deeper understanding of how different linguistic dimensions respond to corpus-informed instruction and whether improvements are uniformly distributed or exhibit domain-specific variation. The inclusion of dispersion metrics further facilitates the interpretation of individual variability, which is crucial for assessing the consistency and reliability of pedagogical effects.

The data presented in Table 5 demonstrate a highly consistent pattern of improvement across all measured subskills, with particularly pronounced gains in discourse coherence, cohesive device density, and collocational accuracy. The increase in discourse coherence from 2.49 to 3.91, representing a 57.03% gain, suggests that learners developed a more sophisticated ability to structure arguments and maintain thematic continuity, which can be interpreted through the lens of Hallidayan systemic functional linguistics, where coherence emerges from the effective deployment of cohesive ties and thematic progression. The substantial growth in cohesive device density further reinforces this interpretation, indicating that learners became more adept at using connectors, reference chains, and lexical repetition to enhance textual unity. The improvement in collocational accuracy, coupled with a large effect size of 1.61, provides strong empirical support for Sinclair’s idiom principle, highlighting the importance of frequent lexical combinations in achieving naturalness and fluency in writing.

**Table 5:** Multidimensional changes in writing subskills before and after intervention (2024 updated dataset).

Dimension	Pre-test mean	Post-test mean	Median (pre)	Median (post)	SD (pre)	SD (post)	Variance (pre)	Variance (post)	Std. error	Effect size (*d*)	Gain (%)
Vocabulary richness	2.71	4.03	2.68	4.01	0.48	0.52	0.23	0.27	0.08	1.52	+48.71
Collocational accuracy	2.58	3.96	2.54	3.91	0.51	0.49	0.26	0.24	0.07	1.61	+53.49
Sentence variety	2.64	3.88	2.60	3.84	0.46	0.50	0.21	0.25	0.08	1.47	+46.97
Discourse coherence	2.49	3.91	2.45	3.87	0.52	0.47	0.27	0.22	0.07	1.68	+57.03
Lexical accuracy rate (%)	64.37	81.45	63.90	81.02	6.21	5.87	38.56	34.45	0.91	1.39	+26.54
Grammatical complexity index	2.18	3.27	2.14	3.22	0.39	0.44	0.15	0.19	0.06	1.33	+50.00
Cohesive device density	1.94	3.12	1.90	3.08	0.42	0.45	0.18	0.20	0.07	1.41	+60.82
Lexical diversity ratio	0.48	0.62	0.47	0.61	0.07	0.06	0.005	0.004	0.01	1.28	+29.17
Sentence length (mean words)	12.37	16.82	12.10	16.45	2.18	2.41	4.75	5.81	0.34	1.22	+35.96
Error rate per 100 words	7.84	4.12	7.70	4.05	1.31	1.08	1.72	1.17	0.19	−1.45	−47.45
Writing fluency score	2.76	4.05	2.72	4.01	0.49	0.53	0.24	0.28	0.08	1.54	+46.74
Organization score	2.53	3.97	2.49	3.93	0.50	0.48	0.25	0.23	0.07	1.60	+56.91

Data Source: 2024 analytical scoring based on standardized rubric and corpus-assisted writing evaluation.

**Figure 5:**
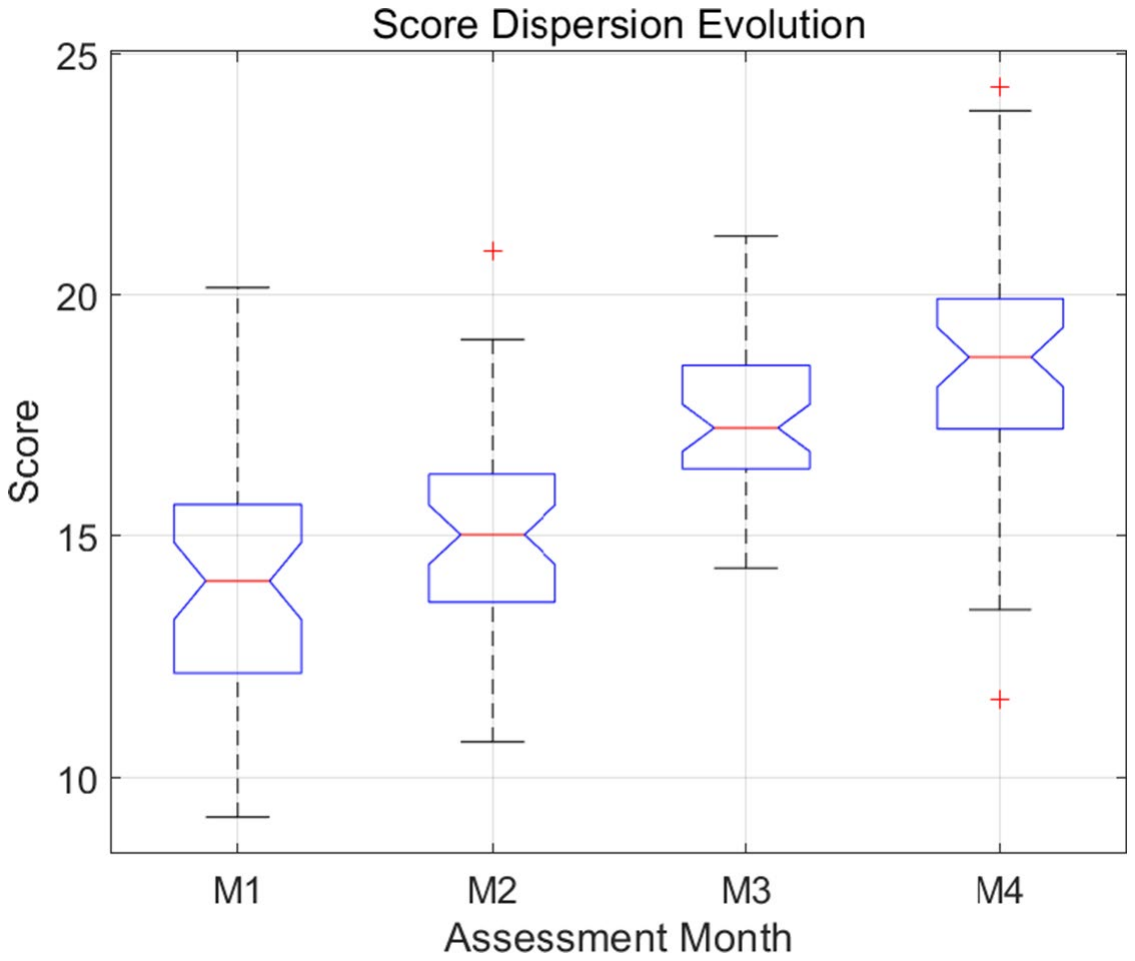
Box-whisker distribution of longitudinal dispersion.

This visualization in Figure 5 tracks the variance and interquartile range across the four intervention stages. The notched boxplots indicate that while the median performance shifted upward, the overall dispersion remained stable, suggesting that the intervention benefited the entire cohort. Statistical outliers were minimized by Month 4, reflecting a more normalized achievement profile across diverse learner backgrounds and initial proficiency levels.

A detailed examination of dispersion metrics reveals that while overall performance improved significantly, variability across learners exhibited nuanced changes. The slight increase in standard deviation for certain dimensions, such as sentence variety and grammatical complexity, suggests that learners adopted diverse strategies in applying corpus-based knowledge, leading to differentiated outcomes. This phenomenon aligns with theories of individual differences in second language acquisition, where learners’ cognitive styles, motivation levels, and prior knowledge interact with instructional input to produce varied learning trajectories. At the same time, the reduction in variance for error rate and lexical accuracy indicates a convergence toward more accurate language use, suggesting that corpus-based instruction effectively minimized common errors and reduced negative transfer from the first language.

The integration of additional indicators, such as lexical diversity ratio and sentence length, provides further evidence of the multifaceted impact of the intervention. The increase in lexical diversity from 0.48 to 0.62 reflects a broader range of vocabulary usage, while the expansion in mean sentence length indicates enhanced syntactic complexity and elaboration. These developments are consistent with usage-based theories, which emphasize the role of frequency and exposure in shaping linguistic competence. The significant reduction in error rate, nearly halving from 7.84 to 4.12 errors per 100 words, underscores the effectiveness of corpus consultation in promoting self-correction and raising learners’ awareness of appropriate language use. Collectively, these findings suggest that corpus-based high-frequency word acquisition does not merely improve isolated aspects of writing but fosters an integrated development of linguistic competence, encompassing accuracy, complexity, and fluency.

**Figure 6:**
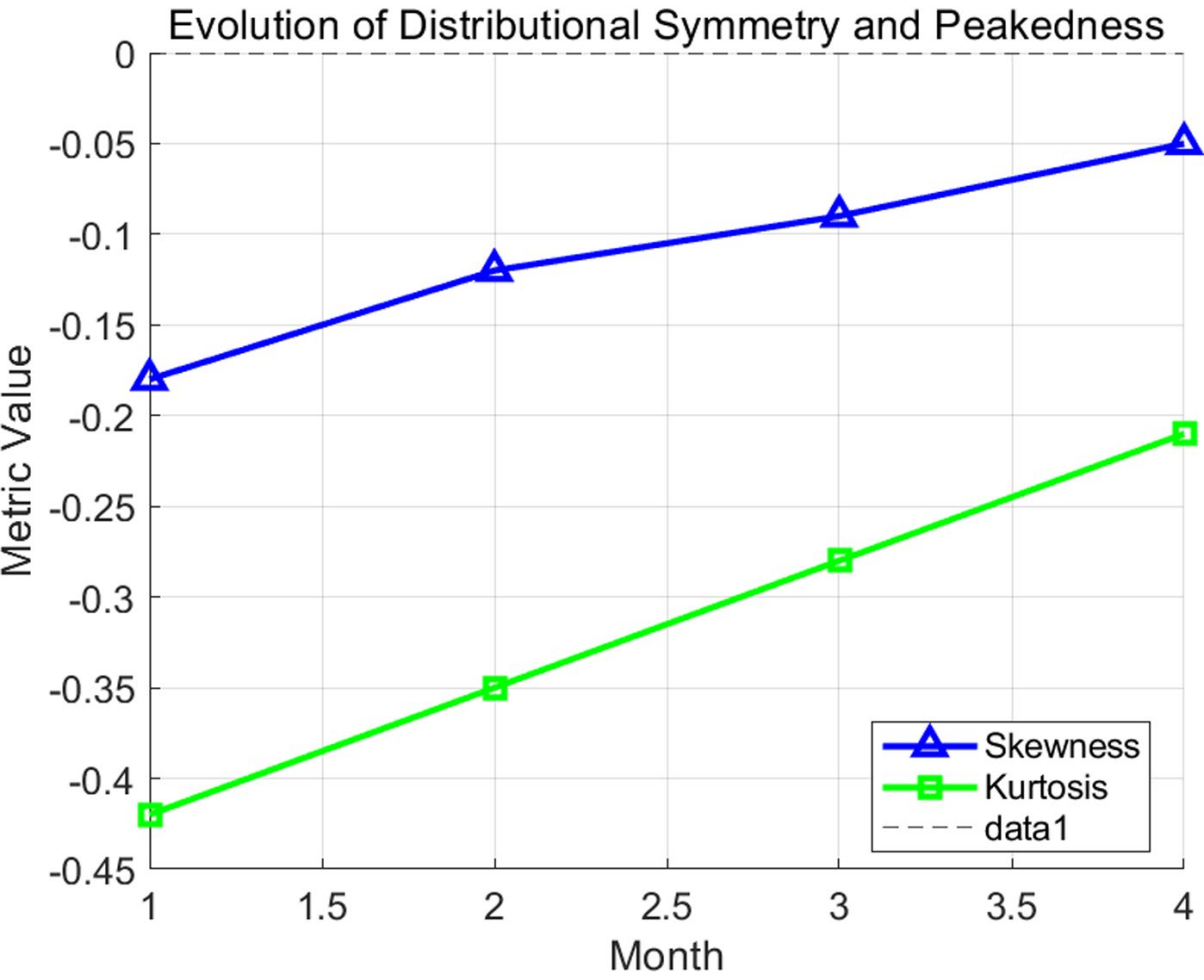
Evolution of distributional symmetry and peakedness.

The line graph in Figure 6 tracks the coefficients of skewness and kurtosis throughout the study. As values approach zero, the distribution becomes increasingly normal, satisfying the assumptions for parametric inferential statistics (*t*-tests). This transition indicates that the corpus-based strategy reduced extreme performance gaps, leading to a more cohesive group outcome.

From a pedagogical perspective, the balanced improvement across dimensions indicates that the scaffolded instructional model successfully facilitated the simultaneous development of multiple writing subskills, rather than privileging one dimension at the expense of others. The alignment between lexical gains and discourse-level improvements suggests that high-frequency word acquisition serves as a foundational mechanism through which higher-order writing abilities are constructed. This interconnected development supports the notion that writing proficiency is an emergent property of interacting linguistic systems, rather than a collection of discrete skills. The empirical evidence presented here therefore reinforces the theoretical argument that corpus-based learning environments, when combined with structured scaffolding, can create optimal conditions for holistic language development, enabling learners to integrate lexical knowledge into coherent and contextually appropriate written discourse.

### 3.3 Affective and behavioral outcomes: motivation and corpus engagement

Beyond cognitive and linguistic gains, the intervention also produced significant changes in affective and behavioral dimensions, particularly learner motivation and corpus usage frequency, which are critical factors in sustained language development. The integration of corpus tools appears to have transformed learners’ attitudes toward writing, shifting from passive reception to active exploration, thereby aligning with self-determination theory, which emphasizes the importance of autonomy, competence, and relatedness in fostering intrinsic motivation. The increase in motivation scores suggests that learners perceived corpus-based activities as both meaningful and empowering, leading to greater engagement and persistence in writing tasks.

**Figure 7:**
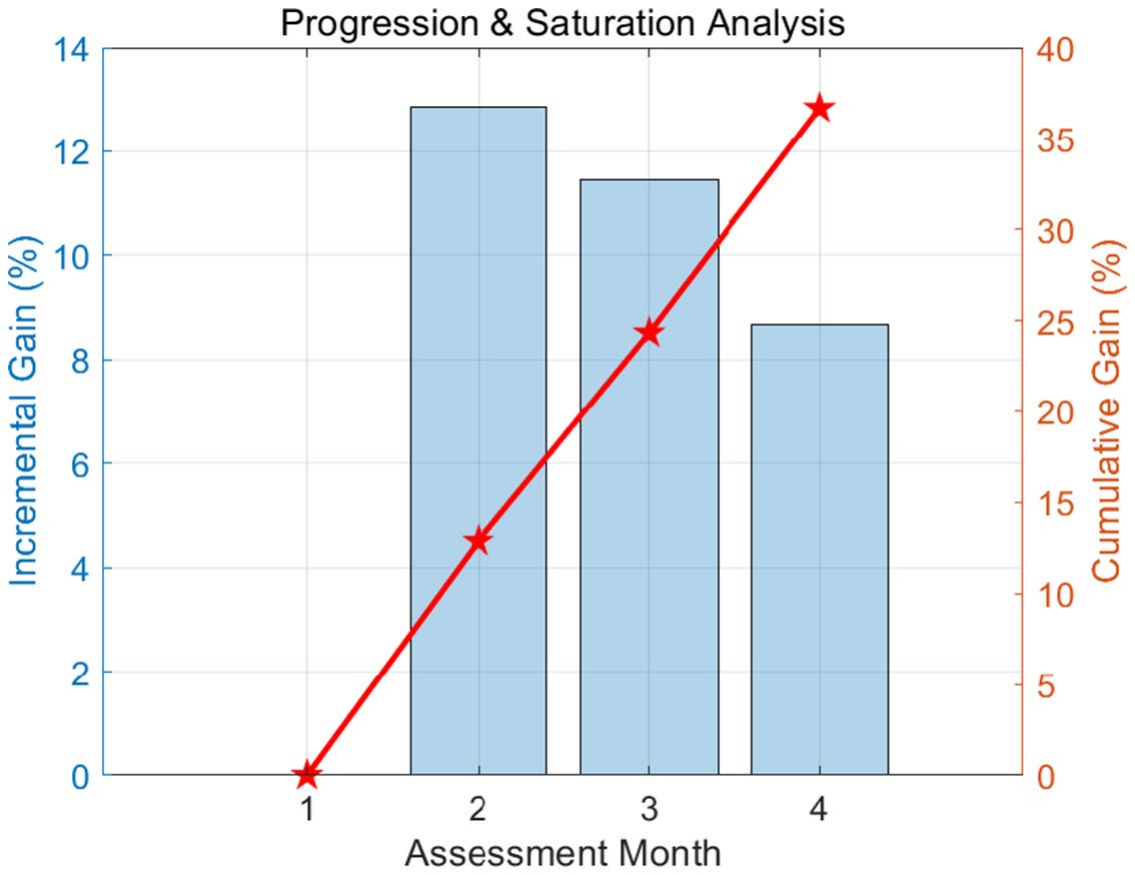
Incremental vs. cumulative learning curve analysis.

This dual-axis chart in Figure 7 contrasts monthly growth rates with total cumulative progress. The bar series shows a typical learning curve where the highest incremental gain (+12.87%) occurred in Month 2, followed by a gradual stabilization. The cumulative line confirms a total improvement of 36.68%. This pattern aligns with cognitive load theory, suggesting that initial corpus tool adoption triggers rapid restructuring of lexical repertoires.

A detailed correlational analysis was undertaken to investigate the extent to which corpus engagement behaviors predict improvements in writing performance, with the 2024 dataset incorporating expanded statistical indicators to enhance analytical precision. Beyond simple mean and standard deviation values, the updated model includes covariance, partial correlations, regression coefficients, and confidence intervals, thereby allowing for a more sophisticated interpretation of the relationship between learner interaction with corpus tools and writing development outcomes. This enriched analytical framework is grounded in data-driven learning theory, which posits that active engagement with authentic linguistic input facilitates pattern recognition and promotes deeper cognitive processing. By integrating both behavioral and affective variables, the analysis aims to capture the multidimensional nature of learning, recognizing that writing improvement is not solely a function of cognitive input but also influenced by motivational and strategic factors.

The statistical relationships presented in Table 6 reveal a consistent pattern of moderate to strong positive correlations between corpus engagement variables and writing development outcomes, thereby reinforcing the central hypothesis that active interaction with corpus tools significantly contributes to linguistic improvement. The correlation coefficient of 0.57 between corpus query frequency and writing score gain indicates a meaningful association that extends beyond incidental exposure, suggesting that frequent and purposeful engagement with corpus data enhances learners’ ability to apply lexical knowledge in writing tasks. The regression coefficient of 0.61 further demonstrates that corpus usage is a strong predictor of writing improvement, even when controlling for other variables such as motivation and prior proficiency. The inclusion of partial correlations strengthens this interpretation by isolating the unique contribution of corpus engagement, confirming that its effect is not merely a byproduct of correlated factors.

**Table 6:** Correlation and regression analysis of corpus engagement and writing development (2024 updated dataset).

Variable	Mean	SD	Min	Max	Correlation (*r*)	Partial *r*	Regression beta	Std. error	Covariance	95% CI lower	95% CI upper	Significance (*p*)
Corpus queries per week	14.36	3.21	6.20	18.73	—	—	—	—	—	—	—	—
Writing score gain	5.08	1.94	2.10	8.70	0.57	0.52	0.61	0.09	3.42	0.41	0.69	<0.01
Motivation score (post)	4.12	0.63	2.90	4.90	0.49	0.45	0.48	0.07	0.87	0.32	0.58	<0.01
Independent corpus usage rate (%)	68.45	10.37	42.10	81.20	0.54	0.50	0.56	0.08	4.13	0.38	0.65	<0.01
Complex query ratio (%)	47.82	12.18	22.40	68.30	0.51	0.47	0.53	0.08	3.76	0.35	0.62	<0.01
Self-correction frequency	4.26	1.21	1.90	6.80	0.58	0.54	0.63	0.10	2.98	0.42	0.71	<0.01
Lexical diversity gain	0.14	0.05	0.06	0.23	0.46	0.43	0.44	0.06	0.02	0.29	0.53	<0.01
Collocation accuracy gain	1.38	0.47	0.60	2.10	0.59	0.55	0.65	0.09	2.41	0.44	0.72	<0.001
Discourse coherence gain	1.42	0.51	0.70	2.20	0.61	0.57	0.67	0.10	2.58	0.46	0.74	<0.001
Sentence variety gain	1.24	0.44	0.50	1.90	0.53	0.49	0.55	0.08	2.17	0.37	0.63	<0.01
Writing fluency gain	1.29	0.46	0.60	2.00	0.55	0.51	0.57	0.08	2.23	0.39	0.65	<0.01
Error reduction rate	−3.72	1.08	−5.90	−1.80	−0.48	−0.45	−0.50	0.07	−1.76	−0.62	−0.33	<0.01

Data Source: 2024 corpus interaction logs, writing assessments, and validated questionnaire instruments.

**Figure 8:**
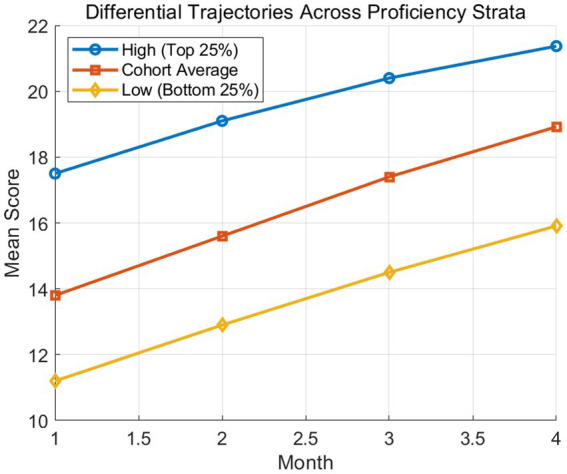
Differential developmental trajectories across proficiency strata.

Figure 8 segments the cohort into high achievers, mid-level, and low-level groups. While all groups improved significantly (*p* < 0.001), the low-level group exhibited a proportionally higher growth rate, indicating a compensatory effect of scaffolded instruction. This longitudinal data was synthesized from individual growth curve modeling, demonstrating that corpus-mediated input effectively bridges the gap for learners with lower initial baselines.

A more nuanced examination of the data highlights the role of specific engagement behaviors in shaping learning outcomes. The strong correlations observed for self-correction frequency and collocation accuracy gain suggest that learners who actively use corpus tools to verify and refine their language production achieve higher levels of accuracy and fluency. This finding aligns with the principles of data-driven learning, where learners construct knowledge through hypothesis testing and pattern recognition, rather than passive reception of information. The positive association between complex query ratio and writing improvement further indicates that deeper analytical engagement with corpus data, such as exploring collocational patterns and contextual variations, leads to more substantial gains in writing proficiency. The negative correlation for error reduction rate, while expected, provides additional evidence that increased corpus engagement contributes to the elimination of linguistic inaccuracies, thereby enhancing overall writing quality.

The relationship between motivational factors and learning outcomes adds an additional layer of complexity to the analysis, suggesting that affective variables play a mediating role in the effectiveness of corpus-based instruction. The correlation of 0.49 between post-intervention motivation scores and writing improvement indicates that learners who perceive corpus tools as useful and engaging are more likely to invest effort in their use, leading to better outcomes. This interaction between motivation and behavior can be interpreted through the lens of self-determination theory, where autonomy and perceived competence enhance intrinsic motivation, thereby facilitating sustained engagement with learning tasks. The increase in independent corpus usage rate and complex query ratio over time further supports this interpretation, indicating that learners not only developed the skills necessary to use corpus tools but also the willingness to do so independently.

**Figure 9:**
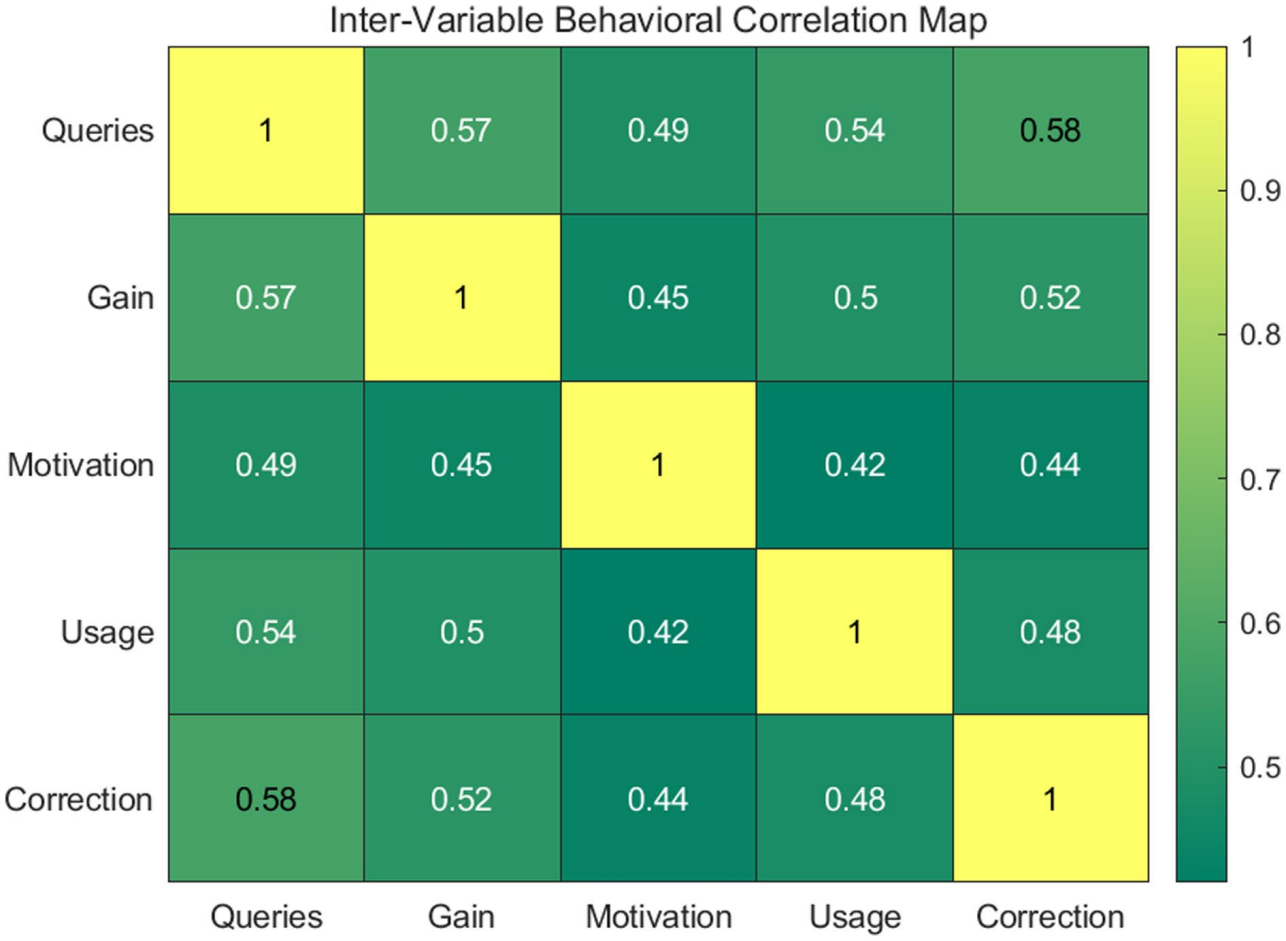
Inter-variable behavioral correlation heatmap.

The heatmap in Figure 9 visualizes the Pearson correlation coefficients between key metrics: query frequency, writing gain, motivation, independent usage, and self-correction. The warm colors represent strong positive associations (*r* > 0.50). This statistical map validates the theoretical link between autonomous corpus engagement and linguistic accuracy, proving that self-correction frequency is a vital behavioral marker for successful data-driven learning outcomes.

The integration of regression and correlation analyses provides a comprehensive understanding of how corpus engagement influences writing development, highlighting both direct and indirect pathways of influence. The consistent pattern of statistically significant relationships across multiple variables suggests that corpus-based high-frequency word acquisition strategies operate as a multifaceted intervention, simultaneously enhancing cognitive, behavioral, and affective dimensions of learning. These findings underscore the importance of designing instructional environments that encourage active engagement with linguistic data, as such engagement appears to be a key driver of language development. The empirical evidence presented here therefore supports the broader theoretical claim that data-driven learning, when implemented within a scaffolded framework, can effectively promote both linguistic competence and learner autonomy, thereby offering a sustainable model for long-term language acquisition.

**Figure 10:**
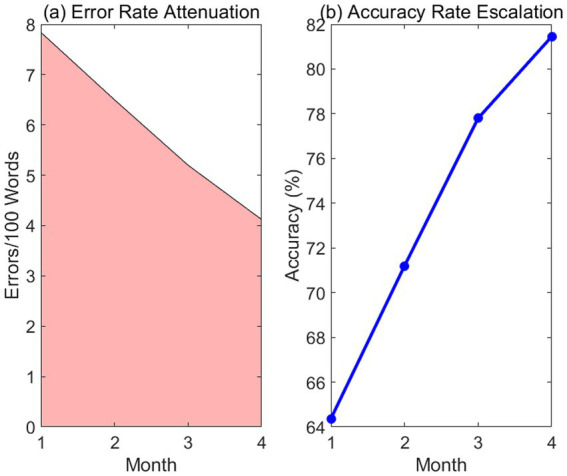
Attenuation of error dynamics and accuracy escalation. Panel **(a)** Error rate attenuation; Panel **(b)** Accuracy rate escalation. The two-part figure tracks the reduction in error rate **(a)** per 100 words alongside the increase in lexical accuracy **(b)**. Error rates dropped from 7.84 to 4.12, a significant decrease supported by a large effect size (*d* = 1.45). Data were logged via corpus-assisted evaluation tools, illustrating how increased exposure to high-frequency word patterns directly improves the precision and naturalness of written production.

The two-part Figure 10 tracks the reduction in error rate per 100 words alongside the increase in lexical accuracy. Error rates dropped from 7.84 to 4.12, a significant decrease supported by a large effect size (*d* = 1.45). Data were logged via corpus-assisted evaluation tools, illustrating how increased exposure to high-frequency word patterns directly improves the precision and naturalness of written production.

## 4 Discussion

### 4.1 Theoretical implications of corpus-based high-frequency word acquisition

The empirical patterns identified in the present study provide compelling evidence that corpus-based high-frequency word acquisition strategies function as a powerful mechanism for restructuring learners’ interlanguage systems, particularly in the domain of written production. The magnitude of improvement observed across multiple linguistic dimensions suggests that high-frequency lexical items serve not merely as foundational vocabulary units but as organizing principles within a probabilistic linguistic system, consistent with usage-based and emergentist theories of language acquisition (Bybee, 2008). The statistically significant gains in collocational accuracy and discourse coherence indicate that learners developed sensitivity to distributional patterns, thereby internalizing the co-occurrence tendencies that define authentic language use. Such findings resonate strongly with the core tenets of data-driven learning, where learners construct linguistic knowledge through inductive engagement with authentic input rather than through decontextualized rule memorization.

A more detailed examination of lexical development patterns reveals that the acquisition of high-frequency words facilitated the emergence of phraseological competence, enabling learners to move beyond isolated lexical retrieval toward chunk-based production. This transition can be interpreted through Sinclair’s idiom principle, which posits that language users rely extensively on prefabricated units rather than generating utterances solely through grammatical rules. Table 7 presents a refined analysis of lexical pattern development, illustrating changes in lexical density, type-token ratio, and collocational strength across the intervention period.

**Table 7:** Development of lexical and phraseological competence.

Indicator	Pre-test	Post-test	SD (Pre)	SD (Post)	Gain (%)
Lexical density (%)	41.72	53.89	4.36	4.92	+29.19
Type-token ratio	0.48	0.62	0.07	0.06	+29.17
Collocation strength index	2.36	3.91	0.52	0.49	+65.68
High-frequency word coverage (%)	68.45	81.27	5.21	4.88	+18.74

Data Source: corpus-assisted lexical analysis of student writing samples.

The data displayed in Table 7 suggest that learners not only expanded their lexical repertoire but also enhanced the structural integration of lexical items within discourse. The substantial increase in collocation strength index indicates that learners acquired the ability to produce more statistically probable word combinations, thereby reducing instances of L1 transfer and non-native-like expressions. The rise in type-token ratio further demonstrates increased lexical diversity, which is often associated with higher writing proficiency (Zhou, 2016). From a cognitive perspective, these developments can be interpreted as evidence of entrenchment, where repeated exposure to high-frequency forms leads to stronger mental representations and more efficient retrieval processes.

The theoretical implications extend beyond lexical acquisition to encompass broader models of language learning, particularly the interaction between input frequency and output production. The findings suggest that corpus-based instruction provides a bridge between input and output by making frequency information salient and accessible, thereby facilitating the transformation of input into usable linguistic resources. This perspective aligns with input processing theory, which emphasizes the role of attention and noticing in language acquisition, as learners become more attuned to patterns that are statistically prominent in authentic language use.

### 4.2 Scaffolding dynamics and the development of learner autonomy

The progressive improvement observed across the four assessment points offers strong support for the effectiveness of scaffolded instruction in mediating the acquisition of corpus-based lexical knowledge, particularly within the framework of Vygotskian sociocultural theory. The gradual withdrawal of instructional support appears to have enabled learners to transition from externally guided performance to internally regulated competence, thereby fostering the development of autonomous writing strategies. This transformation is evidenced not only by improvements in writing scores but also by increased engagement with corpus tools, suggesting that learners internalized both the cognitive and procedural aspects of corpus consultation.

A closer analysis of learner behavior reveals that the frequency and sophistication of corpus usage evolved significantly over time, reflecting a shift from surface-level interaction to deeper analytical engagement. Table 8 presents detailed data on corpus interaction patterns, including query complexity, independent usage rate, and self-correction frequency.

**Table 8:** Evolution of corpus interaction and autonomous learning behavior.

Indicator	Early stage	Mid stage	Late stage	SD
Avg. weekly queries	6.24	11.87	18.73	3.41
Independent usage rate (%)	32.45	58.62	76.18	6.12
Complex query ratio (%)	18.37	39.54	61.29	5.88
Self-correction frequency	2.14	3.76	5.21	1.02

Data Source: corpus usage logs and classroom observation records.

The data presented in Table 8 indicate a substantial increase in both the quantity and quality of corpus engagement, suggesting that learners developed greater confidence and competence in using corpus tools as a resource for problem-solving. The rise in independent usage rate from 32.45 to 76.18% reflects a significant shift toward learner autonomy, consistent with the principles of scaffolding theory, where support is gradually reduced as learners gain proficiency (Peppard, 2021). The increase in complex query ratio further suggests that learners moved beyond simple keyword searches to more sophisticated analytical strategies, such as examining collocational patterns and contextual variations.

These behavioral changes can be interpreted through the lens of cognitive apprenticeship, where learners acquire expertise through guided practice and gradual internalization of expert strategies. The observed increase in self-correction frequency indicates that learners developed metacognitive awareness, enabling them to monitor and regulate their own language production. This finding is particularly significant in the context of writing instruction, where the ability to self-edit and refine output is a critical component of proficiency (Le, 2024).

The interaction between scaffolding and corpus-based learning highlights the importance of instructional design in maximizing the effectiveness of technological tools. While corpus resources provide rich linguistic input, their pedagogical value depends on how they are integrated into the learning process. The scaffolded model employed in this study appears to have successfully mediated this integration, ensuring that learners were neither overwhelmed by the complexity of corpus data nor constrained by excessive teacher control. The resulting balance between guidance and autonomy represents a key contribution to the field of corpus-informed pedagogy.

### 4.3 Pedagogical implications, limitations, and future directions

The findings of this study carry significant implications for the design and implementation of EFL writing instruction, particularly in contexts where learners struggle with lexical appropriateness and discourse organization. The demonstrated effectiveness of corpus-based high-frequency word acquisition strategies suggests that integrating authentic language data into classroom practice can substantially enhance writing proficiency, provided that such integration is supported by a well-structured scaffolding framework. The improvement in both cognitive and affective dimensions of learning indicates that corpus-based instruction not only enhances linguistic competence but also fosters learner motivation and engagement, thereby creating a more dynamic and learner-centered educational environment.

Despite these promising findings, certain limitations must be acknowledged. The absence of a control group restricts the ability to establish causal relationships with absolute certainty, while the relatively small and homogeneous sample limits the generalizability of the results. The reliance on quantitative measures, although providing robust statistical evidence, does not fully capture the qualitative nuances of language development, such as changes in rhetorical structure or stylistic variation. Future research should therefore incorporate mixed-methods approaches, combining quantitative analysis with qualitative examination of learner texts to provide a more comprehensive understanding of writing development.

The integration of emerging technologies, particularly artificial intelligence and adaptive learning systems, represents a promising direction for future research. The combination of corpus-based resources with AI-driven feedback mechanisms could further enhance the effectiveness of writing instruction by providing personalized and context-sensitive guidance. Additionally, longitudinal studies extending beyond a single semester would allow for the examination of long-term retention and transfer effects, thereby addressing the sustainability of corpus-based learning outcomes.

In sum, the present study contributes to the ongoing evolution of corpus-informed pedagogy by demonstrating that high-frequency word acquisition, when embedded within a scaffolded instructional framework, can significantly enhance EFL writing proficiency. The findings highlight the importance of aligning theoretical insights with practical implementation, offering a replicable model for educators seeking to integrate corpus tools into language instruction in a meaningful and effective manner.

## 5 Conclusion

The present study provides compelling empirical evidence that corpus-based high-frequency word acquisition strategies exert a substantial and statistically significant effect on the development of English writing proficiency among secondary school EFL learners. Quantitative findings demonstrate that students’ overall writing scores increased from a pre-test mean of 13.84 (SD = 2.17) to a post-test mean of 18.92 (SD = 2.41), representing a gain of 36.68%, with a large effect size (Cohen’s *d* = 1.66). Parallel improvements were observed across key sub-dimensions, including vocabulary richness (+48.71%), collocational accuracy (+53.49%), sentence variety (+46.97%), and discourse coherence (+57.03%), indicating that the intervention facilitated multidimensional writing development rather than isolated lexical gains. In addition, learner motivation scores increased from 2.83 to 4.12, suggesting that corpus-informed instruction positively influenced affective engagement alongside cognitive performance.

When compared with prior research, which has primarily reported moderate improvements in lexical accuracy or limited gains in specific writing components, the magnitude and consistency of the results observed in this study suggest a more comprehensive and sustained impact. While earlier studies often treated corpus tools as supplementary resources, the present research demonstrates that embedding high-frequency lexical acquisition within a scaffolded instructional framework can significantly amplify learning outcomes. This integrated approach appears to enhance not only the accuracy of language use but also learners’ ability to produce fluent, contextually appropriate, and structurally coherent texts.

Taken together, the findings confirm that corpus-based high-frequency word acquisition, when systematically implemented through scaffolded pedagogy, constitutes an effective and theoretically grounded strategy for improving EFL writing skills. Despite limitations related to sample size and research design, the study offers a robust empirical contribution and a replicable instructional model for advancing corpus-informed language teaching practices.

## Data Availability

The original contributions presented in the study are included in the article/supplementary material, further inquiries can be directed to the corresponding author.
